# Postoperative Metabolic, Inflammation, and Atrial Fibrillation Control: a Relentless Battle

**DOI:** 10.21470/1678-9741-2018-0404

**Published:** 2019

**Authors:** Gibran Roder Feguri, Anna Carolina Franco

**Affiliations:** 1 Cardiology and Cardiovascular Surgery Department, General University Hospital, Universidade de Cuiabá (HGU-UNIC), Cuiabá, MT, Brazil.; 2 Cardiovascular Surgery Department, General University Hospital, Universidade de Cuiabá (HGU-UNIC), Cuiabá, MT, Brazil.

Trauma from cardiovascular surgery results in a well-known metabolic and inflammatory response syndrome, in addition to insulin resistance (IR) and subsequent hyperglycemia. This unfavorable "metabolic status" can last for approximately three weeks or more. Prolonged preoperative fasting may worsen this syndrome, and hyperglycemia is an independent factor for worse prognosis in patients undergoing cardiovascular surgery, leading to prolonged hospital stay, higher infection rates and increased postoperative morbidity and mortality^[1,2]^.

In spite of its significant benefits and technological innovations, cardiopulmonary bypass plays an important role in the development of this syndrome as it causes the release of proinflammatory cytokines and other chemical mediators that, in high concentrations, are associated with organ dysfunction, coagulopathy, ischemia, and adverse clinical outcomes^[2]^.

Postoperative atrial fibrillation (POAF) is a frequent complication of cardiovascular surgery, especially with the use of cardiopulmonary bypass, although it features multifactorial triggers. It has been associated with inflammation and can cause severe discomfort, as well as increased chance of thromboembolic events, postoperative morbidity and mortality and percentage of intensive care unit and hospital readmission^[3]^.

Studies have shown that fasting abbreviation with carbohydrate-rich fluids improves IR and promotes glycemic control, reduces the incidence of postoperative nausea and vomiting, and reduces lean body mass loss-with subsequent increase in postoperative muscle strength. In addition, it improves immune function maintenance in post-surgical trauma and reduces length of hospital stay^[2,4]^.

In Brazil, the first study reporting preoperative fasting abbreviation with carbohydrate (CHO) intake in cardiovascular surgery was published in 2012^[2]^, in which the ACERTO protocol (ACEleração da Recuperação Total Pós-Operatória; an example of a national multimodal protocol) was used^[4]^. The intervention group showed favorable clinical and metabolic outcomes and shorter hospital and intensive care unit length of stay; however, there was no change in IR in the sample studied.

On the other hand, omega-3 polyunsaturated fatty acids (ω-3 PUFA) are no longer exclusive to clinical cardiology. In the last decade, ω-3 PUFA have become an important ally in postoperative inflammation and metabolic control in cardiovascular surgery, as it is directly related to a reduction in proinflammatory cytokines and incidence of postoperative arrhythmia, particularly postoperative atrial fibrillation. The protective effects of this type of fish oil have also been observed in critically ill patients in the intensive care unit, usually in parenteral nutrition regimens^[5,6]^.

Berger et al.^[5]^ conducted a novel study in which they analyzed 28 patients undergoing coronary artery bypass grafting (with or without valve replacement), who received 3 infusions of 0.2 g/kg of either ω-3 PUFA or placebo at 12 hours and 2 hours before surgery and immediately after surgery. Serial blood sampling and intraoperative atrial biopsy were carried out to assess oil absorption by the cell membranes. Results showed that perioperative infusions significantly increased the concentration of ω-3 PUFA in platelet membrane and atrial tissue within 12 hours of the first infusion and significantly lowered clinical and biological signs of inflammation. In a recent meta-analysis (14 studies) on the use of ω-3 PUFA, Wang et al.^[6]^ showed promising results, with a lower incidence of POAF (RR 0.84 [95% CI 0.73-0.98], *P*=0.03). These results, however, were only for coronary artery bypass grafting (0.68 [0.47-0.97], *P*=0.03); none of the other types of surgery analyzed showed similar results.

Moreover-and who would have thought-, studies have shown the benefits of vitamin C (ascorbic acid) in preventing atrial fibrillation in the postoperative period of cardiovascular surgery. A recent meta-analysis of 15 clinical trials for the prevention of atrial fibrillation showed that in 9 of the studies vitamin C lowered the incidence of postoperative atrial fibrillation (RR = 0.56 [95% CI 0.47-0.67], *P*<0.01), and reduced the length of hospital stay by 12.6% (95% CI 8.4-16.8%, *P*<0.01) and in the intensive care unit by 8.0% (95% CI 3.0-13.0%, *P*<0.01). However, the authors found significant differences in the possible effects of vitamin C to preventing postoperative atrial fibrillation and they conclude that more research is needed to define protocols with a precise dosage as well as to identify which groups of ideal patients could benefit the most from its use^[7]^.

These alternatives become interesting in a global setting under constant change and discussion. A case in point is the last year's guideline for perioperative medication published by the European Association for Cardio-Thoracic Surgery (EACTS)^[8]^, which no longer recommends the routine use of corticosteroids to control inflammation and prevent postoperative atrial fibrillation in cardiovascular surgery, while the use of ω-3 PUFA for this purpose is already under discussion.

The constant search for postoperative metabolic and inflammation control in cardiovascular surgery is justifiable, given the great impact on clinical outcomes, the high costs, and the potential benefits that could be achieved with a more adequate control. The original article conducted by Feguri et al.^[9]^, available in this issue, gives the reader an unprecedented association of preoperative fasting abbreviation with CHO and intraoperative infusion of ω-3 PUFA, as well as interesting results showing that the method is safe. The authors also provide a brief review of this topic and highlight the potential for faster postoperative recovery of patients undergoing on-pump coronary artery bypass grafting.
